# Structural Insights into the Interaction of Cytochrome P450 3A4 with Suicide Substrates: Mibefradil, Azamulin and 6′,7′-Dihydroxybergamottin

**DOI:** 10.3390/ijms20174245

**Published:** 2019-08-30

**Authors:** Irina F. Sevrioukova

**Affiliations:** Department of Molecular Biology and Biochemistry, University of California, Irvine, CA 92697-3900, USA; sevrioui@uci.edu

**Keywords:** CYP3A4, mechanism-based inhibitor, crystal structure

## Abstract

Human cytochrome P450 3A4 (CYP3A4) is the most important drug-metabolizing enzyme. Some drugs and natural compounds can act as suicide (mechanism-based) inactivators of CYP3A4, leading to unanticipated drug-drug interactions, toxicity and therapeutic failures. Despite significant clinical and toxicological implications, the mechanism-based inactivation remains incompletely understood. This study provides the first direct insights into the interaction of CYP3A4 with three suicide substrates: mibefradil, an antihypertensive drug quickly withdrawn from the market; a semi-synthetic antibiotic azamulin; and a natural furanocoumarin, 6′,7′-dihydroxybergamottin. Novel structural findings help better understand the suicide substrate binding and inhibitory mechanism, and can be used to improve the predictability of the binding ability, metabolic sites and inhibitory/inactivation potential of newly developed drugs and other chemicals relevant to public health.

## 1. Introduction

Human cytochrome P450 3A4 (CYP3A4) oxidizes over 50% of administered drugs [1], along with natural compounds, some of which can act as inhibitors of CYP3A4 [2]. The mechanism-based (suicide) inhibition (MBI) of CYP3A4 is the most common mechanism that could lead to clinically significant drug-drug interactions (DDIs), toxicity and therapeutic failures [3]. MBI is characterized by NADPH-, time- and concentration-dependent enzyme inactivation due to the formation and covalent attachment of a reactive metabolite(s) to the heme and/or apoprotein. The early elimination of the MBI/DDI potential in drug candidates is crucial [4] but highly challenging due to poor predictability of CYP3A4-ligand interactions. This results from high promiscuity [5] and conformational flexibility of CYP3A4 [6,7,8] and very limited structural information on the substrate association modes. To date, only three crystal structures of CYP3A4 with drug substrates bound in the active site have been reported [6,7,9] and none with suicide inactivators. 

**Figure 1:**
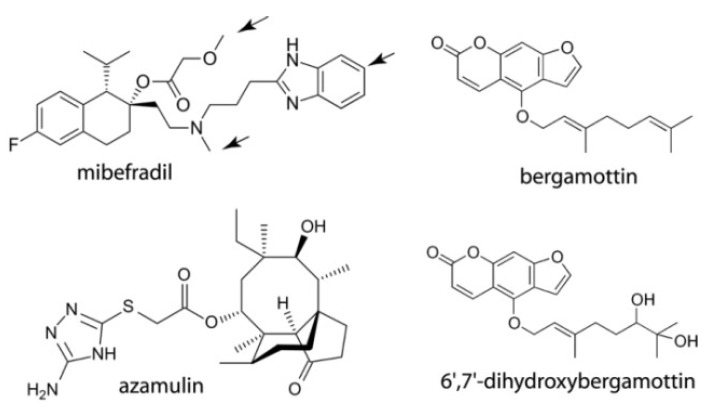
Chemical structures of the investigated compounds. Arrows indicate known sites of metabolism.

To fill this knowledge gap, this study investigated the binding manner of mibefradil, azamulin, bergamottin and 6′,7′-dihydroxybergamottin (DHB) (Figure 1), which are known to act as potent mechanism-based inhibitors of CYP3A4. Mibefradil (or Posicor) is a benzimidazoyl-substituted tetraline, designed as a long-acting T-type calcium channel blocker for the treatment of chronic hypertension. Mibefradil interacts with multiple cytochrome P450 isoforms, but was withdrawn from the market mainly due to its high inhibitory potency for CYP3A4, leading to numerous life-threatening DDIs [10]. Azamulin is a semi-synthetic antibiotic and derivative of pleuromutilin, which failed stage I clinical trials due to poor bioavailability. Azamulin was recently identified as an effective and highly selective inhibitor of CYP3A4 and is currently recommended for use in reaction phenotyping studies instead of less specific ketoconazole [11,12]. Bergamottin and its derivative DHB, on the other hand, are natural furanocoumarins found most abundantly in grapefruits. In addition to several health-promoting effects [13], both compounds mediate food-drug interactions primarily through inhibition of CYP3A4 [14]. Here, the results of spectral investigations are reported, as well as crystal structures of CYP3A4 bound to mibefradil, azamulin and DHB. Our findings help better understand the suicide substrate binding and inhibitory mechanism, and could improve the computational tools for modeling and prediction of the CYP3A4-ligand interactions, which is vital for designing safer and more effective drugs.

## 2. Results and Discussion

### 2.1. Interaction of CYP3A4 with Mibefradil

**Figure 2:**
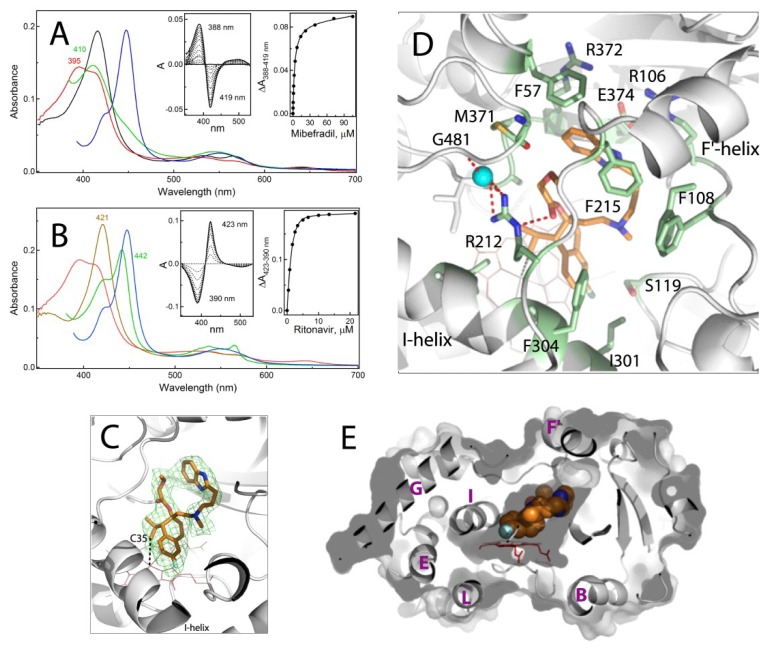
Spectral and structural properties of the CYP3A4-mibefradil complex. (**A**,**B**) Spectral changes observed during equilibrium titrations of CYP3A4 with mibefradil and upon displacement of mibefradil with ritonavir, respectively. In panel (**A**), the spectrum of ligand-free CYP3A4 is in black. In panel (**B**), the spectrum of ritonavir-bound CYP3A4 is in brown. In both panels, the spectra of the CYP3A4-mibefradil complex and its ferrous and ferrous CO-bound forms are in red, green and blue, respectively. In the competitive displacement experiment (panel **B**), the concentration of mibefradil was 120 μM. The left and right insets are the difference spectra and titration plots with hyperbolic fittings, respectively. The derived K_s_ values are given in Table 1. (**C**) The active site of CYP3A4 bound to mibefradil (shown in orange sticks; PDB ID 6OO9). Green mesh is a polder omit electron density map contoured at 3σ level. Simulated annealing omit map for mibefradil is shown in Figure S2A. The labeled C35 atom of mibefradil is the closest to the heme iron (~3.8 Å away). (**D**) Interaction of mibefradil with surrounding residues (shown in green sticks and labeled). Red dotted lines are H-bonds. Cyan sphere is a water molecule. (**E**) A slice through the CYP3A4 molecule showing how well mibefradil (in space-filling representation) fits into the active site cavity. The visible helices are labeled.

Equilibrium titrations of the full-length CYP3A4 were conducted to determine and relate the binding affinity and dissociability of the investigated compounds to their association mode. Mibefradil is a type I ligand that causes a blue shift in the Soret band (Figure 2A). The spectral dissociation constant (K_s_) derived from the titration plot (left inset in Figure 2A) was 3.3 μM, which is ~5-fold higher than the previously reported value [15], possibly due to differences in the CYP3A4 form. Since CYP3A4 precipitates during prolonged dialysis, the dissociation ability was assessed by titrating the ligand-bound protein with ritonavir, a high-affinity inhibitor that easily displaces other type I substrates, such as bromocryptine and midazolam (Figure S1). As seen in Figure 2B, ritonavir could fully replace mibefradil, but its binding affinity was 74-fold lower than for the ligand-free CYP3A4: K_s_^RIT^ of 1.4 μM vs. 0.019 μM [16], respectively. In contrast, only a 3.5-fold decrease in K_s_^RIT^ was observed for the bromocryptine- and midazolam-bound CYP3A4 (Table 1). Thus, mibefradil interacts with CYP3A4 stronger and/or is better protected and has a lower ability to dissociate.

#### 2.1.1. Crystal Structure of the CYP3A4-Mibefradil Complex

Mibefradil inhibits microsomal and recombinant CYP3A4 with IC_50_ of 0.3–2 μM [17], but the chemical nature of its reactive metabolite(s) is still unknown. A previous attempt to elucidate the molecular basis for MBI led to a conclusion that the inactivation of CYP3A4 proceeds through heme destruction rather than covalent modification of the heme or apoprotein [15]. To identify the potential oxidation sites that could lead to bioactivation, CYP3A4 was co-crystallized with mibefradil. The crystal structure was determined to 2.25 Å resolution (Table S1) and contained one drug molecule in the active site (Figure 2C–E). The tetraline moiety is 3.3–3.6 Å above the heme plane, with the propyl C35 atom being the closest to the iron (~3.8 Å away; Figure 2C). The fluorophenyl portion is parallel to the I-helix and partially inserts into a hydrophobic pocket formed by F304, A305 and I301. The benzimidazole moiety provides additional hydrophobic and aromatic interactions with F57, F215 and M371 (Figure 2D). The methoxyacetate functionality, in turn, is H-bonded via the carbonyl oxygen to the R212 guanidine group. This polar interaction is part of the H-bonding network that links the F-F′-loop to the protein core and likely contributes to the inhibitory potency, as the derivatives lacking the methoxyacetate group are weaker inhibitors of CYP3A4 than mibefradil [15,18]. The ligand binding mode is further stabilized by multiple van der Waals interactions.

As seen from Figure 2E and Figure S3A, mibefradil binds compactly and fits into the catalytic cavity without triggering any notable conformational change. However, the compact binding mode may limit its motional freedom. This limitation and the fact that CYP3A4 remains in a resting conformation, tightly locked through the R212-mediated contacts, could explain the low dissociability of mibefradil.

#### 2.1.2. Possible Inhibitory Mechanism of Mibefradil

CYP3A4 metabolizes mibefradil via methoxyacetate and tertiary amine demethylation and hydroxylation of the benzimidazole ring (indicated in Figure 1) rather than the oxidation of the closest to the iron propyl group [10]. Thus, the re-entry or reorientation of mibefradil would be required to allow an access to the primary, non-inhibitory sites of metabolism. Whether the crystallographic binding mode could lead to products capable of escaping the active site is yet to be proven. Even so, based on our and earlier findings [10,15,18], it is plausible to suggest that the crystal structure represents an inhibitory complex, where mibefradil could decrease the CYP3A4 activity in two ways: (i) through formation of a slowly dissociable complex that would prevent other substrate molecules from reaching the catalytic center; and (ii) by producing an alkyl radical intermediate upon a hydrogen abstraction from the C35 atom, which could attack and destroy the heme. The formation of a highly reactive free radical would explain why the C35 oxidation product was not observed.

### 2.2. Interaction of CYP3A4 with Azamulin

**Figure 3:**
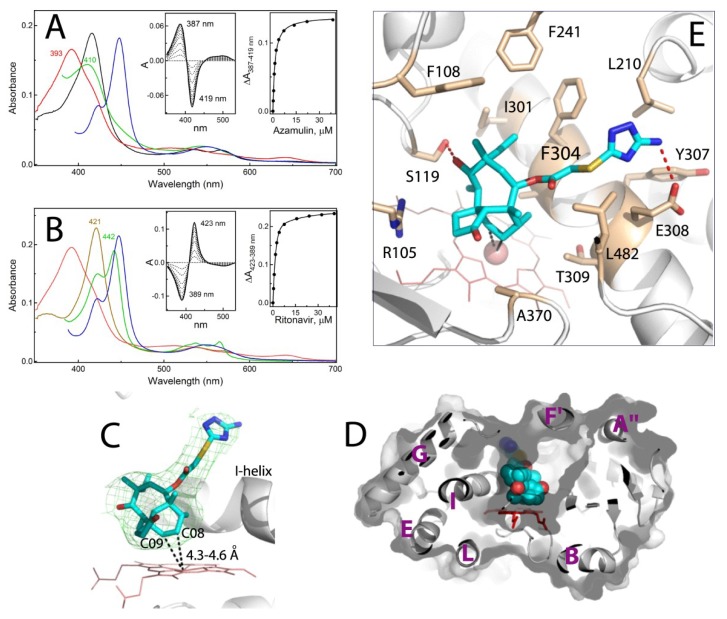
Spectral and structural properties of the CYP3A4-azamulin complex. (**A**,**B**) Spectral changes observed during equilibrium titrations of CYP3A4 with azamulin and upon displacement of azamulin with ritonavir, respectively. In panel (**A**), the spectrum of ligand-free CYP3A4 is in black. In panel (**B**), the spectrum of ritonavir-bound CYP3A4 is in brown. In both panels, the spectra of the CYP3A4-azamulin complex and its ferrous and ferrous CO-bound forms are in red, green and blue, respectively. In the competitive displacement experiment (panel **B**), the concentration of azamulin was 60 μM. The left and right insets are the difference spectra and titration plots with hyperbolic fittings, respectively. The derived K_s_ values are listed in Table 1. (**C**) The binding mode of azamulin (in cyan; PDB ID 6OOA). Green mesh is a polder omit electron density map contoured at 3σ level. Simulated annealing omit map for azamulin is shown in Figure S2B. The closest to the iron C08 and C09 atoms are indicated. (**D**) A slice through the CYP3A4 molecule showing that azamulin (in space-filling representation) extends over the I-helix rather than along the substrate channel. The visible helices are labeled. (**E**) Interaction of azamulin with surrounding residues (shown in beige sticks and labeled). The H-bonds are depicted as red dotted lines.

Azamulin is also a medium affinity type I ligand (K_s_ of 1.7 μM; Figure 3A) which, unlike mibefradil, causes a complete high-spin shift in CYP3A4. The observed absorbance changes suggest that azamulin docks with the bulky pleuromutilin head on, as the heme ligation to the amino-triazolyl nitrogen would lead to a red shift in the Soret band (type II spectral perturbations). Despite the higher affinity for CYP3A4, azamulin was displaced by ritonavir more easily than mibefradil but less easily than other type I ligands (Figure 3B; Table 1). This implies that azamulin is a slowly dissociable ligand that forms fewer/weaker contacts and/or is less protected in the active site than mibefradil.

It should be noted also that K_s_^RIT^ serves as an estimate for the displaced ligand dissociation constant. The fact that the K_s_^RIT^ and K_s_ values derived for mibefradil and azamulin are very close (only 2-fold difference; Table 1) suggests that in the competitive displacement experiments the substrate dissociation is a limiting step, which is only weakly affected by ritonavir.

#### Crystal Structure of CYP3A4 Bound to Azamulin

The crystal structure of the CYP3A4-azamulin complex was determined to 2.52 Å resolution (Table S1) and contains one drug molecule bound to the active site in an extended conformation (Figure 3C). As the spectral data predicted, the pleuromutilin functionality is placed near the heme. The complex could be productive, because two carbon atoms of the hexane ring, C08 and C09, are close enough for oxidation: 4.6 Å and 4.3 Å from the iron, respectively. The thioacetyl linker is stretched above the I-helix and, as a result, the amino-triazolyl end-group points upward rather than toward the substrate channel (Figure 3C,D). This conformation is stabilized by multiple van der Waals contacts and two hydrogen bonds, formed between the hydroxyl group of pleuromutilin’s eight-membered ring and S119 side chain, and between the amino-triazolyl nitrogen and E308 carboxyl (Figure 3E). Importantly, due to steric clashing with the amino-triazolyl moiety, the F-F′-loop (residues 210–217) becomes disordered, leaving the end-portion of azamulin partially solvent exposed (Figure S4). This suboptimal binding mode could explain why azamulin is displaced by ritonavir more easily than mibefradil.

Azamulin was identified as a potent and highly selective competitive and mechanism-based inhibitor of microsomal and recombinant CYP3A4 (IC_50_ of 0.03–0.24 μM) [11]. The pleuromutilin group is thought to undergo metabolic activation [11], but the reactive intermediates are yet to be identified. The crystal structure corroborates the notion that the pleuromutilin is required for MBI, because this functionality is the closest to the heme, mediates the majority of protein-ligand interactions and orients suitably for oxidation. It needs to be tested though whether the C08/9 oxidation products could be reactive or they would have to dissociate and rebind in a distinct orientation to enable bioactivation at other sites. In any case, considering the bulkiness, high hydrophobicity and low dissociability of azamulin, it is plausible to suggest that this compound could effectively inhibit CYP3A4 by crowding/blocking the active site as well.

### 2.3. Interaction of CYP3A4 with Bergamottin and DHB

**Figure 4:**
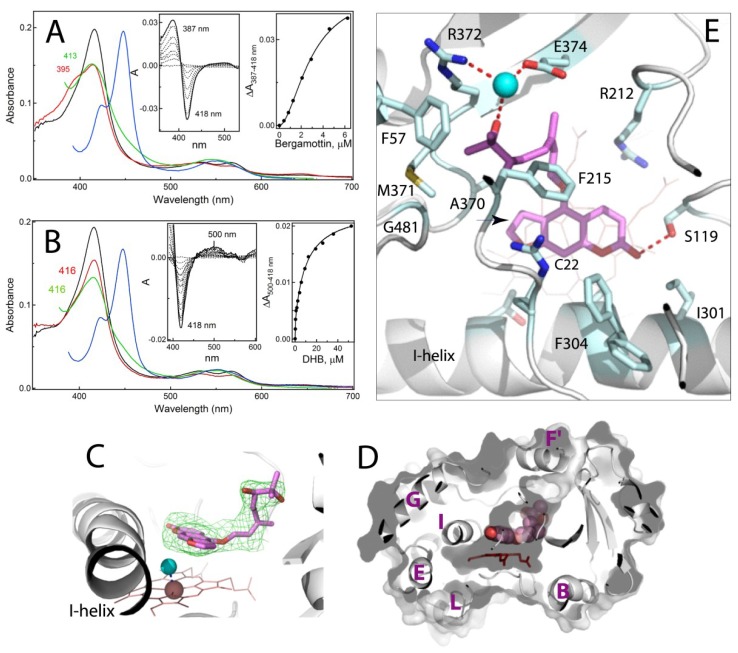
Interaction of CYP3A4 with bergamottin and DHB. (**A**,**B**) Spectral changes observed during equilibrium titration of CYP3A4 with bergamottin and 6′,7′-dihydroxybergamottin (DHB), respectively. The spectra of ligand-free CYP3A4 are in black. The spectra of the CYP3A4-substrate complexes and their ferrous and ferrous CO-bound forms are in red, green and blue, respectively. The left and right insets are the difference spectra and titration plots with sigmoidal and hyperbolic fittings, respectively. The derived K_s_ values are listed in Table 1. (**C**) The binding mode of DHB (in magenta; PDB ID 6OOB). Green mesh is a polder omit electron density map contoured at 3σ level. Simulated annealing omit map for DHB is shown in Figure S2C. Cyan sphere is the heme-bound water molecule. (**D**) A slice through the CYP3A4 molecule showing how DHB (in space-filling representation) orients in the active site cavity. The visible helices are labeled. (**E**) Interaction of DHB with surrounding residues (shown in cyan sticks and labeled). Red dotted lines are H-bonds. Cyan sphere is a water molecule. The furan double bond, a possible bioactivation site, is indicated by an arrow. The C22 atom of psoralen’s phenyl ring, the closest to the heme iron, is labeled.

Bergamottin, but not DHB, causes a partial high-spin shift in CYP3A4 (Figure 4A,B). An increase in DHB concentration leads to a small decrease rather than a shift in the Soret band, meaning that DHB could approach and alter the heme environment without changing the coordination state. Another notable difference was in the shape of the titration curves: sigmoidal for bergamottin and hyperbolic for DHB (right insets in Figure 4A,B). The S_50_ and *n* values (substrate concentration at half-saturation and the Hill coefficient, respectively) derived from the sigmoidal plot suggest that bergamottin binds to CYP3A4 cooperatively and with affinity comparable to those of mibefradil and azamulin (Table 1). The DHB titration curve, in turn, was best fit to a two-site binding hyperbolic equation, indicating that (i) two DHB molecules can simultaneously bind to CYP3A4, and (ii) the occupation of the high affinity site (K_s_ of 0.22 μM) leads to very small changes in the Soret band (~17% of the total absorbance decrease). Another notable feature is the incomplete reduction of bergamottin- and DHB-bound CYP3A4 with sodium dithionite, as evident from the red-shifted absorbance maxima of their ferrous forms: 413 and 416 nm, respectively, versus 410 nm for mibefradil- and azamulin-bound CYP3A4 (compare green spectra in Figure 2A, Figure 3A and Figure 4A,B). Thus, both furanocoumarins seem to limit an access of the reductant to the heme iron. Since the reaction of CYP3A4 with sodium dithionite was carried out under aerobic conditions and was the slowest for the DHB-bound form, the lower than expected 450 nm absorption of the ferrous DHB/CO-bound species (blue spectrum in Figure 4B) could be the consequence of both the incomplete reduction and oxidative damage of the heme. Despite the markedly distinct binding manner, both bergamottin and DHB could be easily displaced by ritonavir (Figure S5), whose binding affinity was only mildly affected (<2-fold decrease; Table 1).

#### 2.3.1. Crystal Structure of the CYP3A4-DHB Complex

We attempted to co-crystallize CYP3A4 with both bergamottin and DHB but succeeded in obtaining only the DHB-bound crystals. The latter structure was solved to 2.2 Å resolution (Table S1) and contains one DHB molecule in the active site (Figure 4C–E). The psoralen ring of DHB is placed 3.6–5.2 Å above the heme (~15° tilt) and parallel to the I-helix, with the carbonyl oxygen H-bonded to the S119 hydroxyl group. As the spectral data predicted, the heme iron remains hexa-coordinate but the water ligand shifts toward the I-helix, altering the Fe-O bond perpendicularity by ~7° (Figure 4C). This subtle perturbation in the heme ligation explains why only small spectral changes could be detected during the equilibrium titrations.

The dihydroxygeranyl group curls along the substrate channel and anchors to its wall through the water-mediated H-bond, linking 7′-OH to the R372 and E374 side chains. F57, F215, A370 and M371 create a hydrophobic environment for the aliphatic portion of the geranyl moiety, whereas R212 and F304 flank the psoralen ring. The F304 side group adopts two alternative conformers: one pointing away and another toward the active site, as in ligand-free CYP3A4 (Figure 4E). Since the DHB site is fully occupied, this conformational heterogeneity is likely caused by steric hindrance with the psoralen ring. Considering the largely different affinities for the two binding sites (Table 1), it is reasonable to conclude that DHB occupies the high affinity site. However, the crystallographic binding mode is non-productive, because the nearest to the iron C22 atom of psoralen’s phenyl ring (indicated in Figure 4E) is too far for oxidation (>5Å).

#### 2.3.2. Possible Mechanism for DHB Bioactivation

Bergamottin and DHB mediate food-drug interactions primarily through reversible and mechanism-based inhibition of CYP3A4 (IC_50_ of 2–23 μM) [14,19,20]. The structure-activity studies on natural furanocoumarins showed that a plain tricyclic ring containing the furan moiety is strictly required for inhibiting CYP3A4 activity [21]. This led to a suggestion that bioactivation of bergamottin and DHB proceeds via epoxidation and opening of the furan ring [22,23]. In the crystal structure, however, the furan double bond (indicated in Figure 4E) is too remote and cannot approach the catalytic center without rotation of the psoralen ring, which might be difficult to achieve due to close proximity of the heme and I-helix.

**Figure 5:**
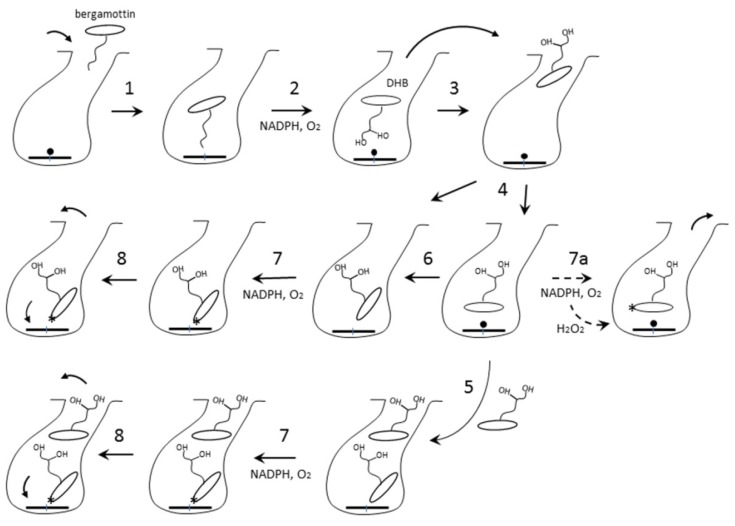
Possible DHB binding and bioactivation mechanism. The parent compound, bergamottin, preferably binds to CYP3A4 with the hydrophobic geranyl group entering the substrate channel (step 1). This binding mode is productive and leads to formation of DHB, which dissociates and re-enters the active site with the psoralen group approaching the heme iron (steps 2–3). DHB could have multiple orientations and two binding sites (steps 4–6). In some binding modes, psoralen’s furan ring will be close enough to the iron to allow enzymatic bioactivation (step 7). Alternatively, the furan double bond can be oxidized by a by-product, hydrogen peroxide (step 7a). The reactive metabolite(s) could attack and destroy the heme or escape the active site and modify the apoprotein (step 8), leading to alterations in conformational dynamics and structural integrity of CYP3A4. The bioactivation site is indicated by an asterisk.

To reconcile our and the previous results [22,23], we propose the following mechanism (Figure 5). Having a more hydrophobic aliphatic chain, bergamottin preferably enters the active site with the geranyl being the closest to the heme iron (step 1), as observed in the 6DWM structure of CYP1A1 [24]. This binding mode is productive and leads to formation of DHB and singly hydroxylated products (step 2). Being more hydrophilic, the oxidation products would dissociate and reenter the active site with the psoralen group approaching the heme (steps 3–4). This orientation could enable association of the second DHB molecule, which may alter the conformation of DHB bound to the high-affinity site (step 5). Occupation of both binding sites would enhance the inhibitory potential, as furanocoumarin dimers are known to inhibit CYP3A4 stronger than DHB [21,25,26]. Nonetheless, the higher-affinity ligands could fully displace DHB and, thus, its metabolic activation would be a prerequisite for the potent inhibition. Among multiple DHB orientations, only some would be suitable for the enzymatic bioactivation of the furan ring (steps 6–7). Alternatively, since CYP3A4 catalysis is highly uncoupled [27], the furan double bond could be oxidized by a by-product, hydrogen peroxide, even when DHB adopts the crystallographic binding mode (step 7a). Upon epoxidation and opening of the furan ring, the radical intermediate could attack the heme (step 8). The epoxide product, in turn, could diffuse out of the active site and modify the apoprotein [22]. This could lead to alterations in conformational dynamics and structural integrity of CYP3A4 and its accelerated degradation [28].

In summary, this study provided the first direct insights into the interaction of human drug-metabolizing CYP3A4 with three structurally diverse suicide substrates: mibefradil, azamulin and DHB. Only minimal conformational adjustments were needed to accommodate these compounds which, instead, were molded or stretched for a better fit and optimization of protein-ligand contacts. In addition to S119 and R212, frequently involved in the ligand binding, three more polar residues, E308, R372 and E372, were identified as important for the formation/stabilization of the substrate-bound complexes. The CYP3A4-DHB structure identified the high-affinity area where furanocoumarins and other small hydrophobic molecules bearing few polar groups could preferably dock. The binding manner of mibefradil and azamulin, on the other hand, suggested that these compounds could exert their inhibitory action not only upon bioactivation of the newly identified or other oxidation sites, but also by forming slowly dissociable complexes that disallow other substrates to access the catalytic site. Together, the spectral and structural data help better understand the suicide substrate binding and inhibitory mechanism, enable more accurate mapping of the CYP3A4 active site, and can be used to improve computational tools for the prediction of the binding ability, metabolic sites and MBI/DDI potential of newly developed medicines and other chemicals relevant to human health.

## 3. Materials and Methods

Mibefradil was obtained from Tocris Bioscience (Minneapolis, MN, USA) azamulin and DHB from Cayman Chemical (Ann Arbor, MI, USA), and bergamottin from Sigma-Aldrich (St. Louis, MO, USA).

### 3.1. Protein Expression and Purification

The codon-optimized full-length and Δ3-22 human CYP3A4 were produced as reported previously [29] and used for the spectral and structural studies, respectively.

### 3.2. Spectral Binding Titrations

Ligand binding to CYP3A4 was monitored in a Cary 300 spectrophotometer at ambient temperatures in 0.1 M phosphate buffer, pH 7.4, containing 20% glycerol and 1 mM dithiothreitol. The investigated compounds were dissolved in dimethyl sulfoxide (DMSO) to 0.2–20 mM concentration and added to a 1.5–2 μM protein solution in small aliquots, with the final solvent concentration <2%. After the addition of each aliquot, the reaction mixture was permitted to stand until no further absorbance changes could be detected, usually less than 20 min. At the end of titrations, the spectra of the ferrous CO-adduct were recorded to ensure that there was no CYP3A4 inactivation during lengthy measurements. Difference spectra were recorded in a separate experiment, where glycerol was omitted and equal amounts of DMSO were added to the reference cuvette to correct for the solvent-induced spectral perturbations. The spectral dissociation constant (K_s_) was derived from the hyperbolic, sigmoidal or quadratic fits to the titration curves using IgorPro (WaveMetrics, Inc., Portland, OR, USA). To assess the ligands’ dissociability, CYP3A4 was saturated with the substrate and then titrated with ritonavir (0.2–5 mM solution in DMSO) to determine how occupation of the active site affects its dissociation constant (K_s_^RIT^). Each titration experiment was repeated three times. The average K_s_ values and standard errors are given in Table 1. The high-spin content was estimated based on the absorbance spectra of ligand-free (100% low-spin) and azamulin-bound CYP3A4 (100% high-spin conversion).

### 3.3. Determination of the X-ray Structures

Δ3-22 CYP3A4 was co-crystallized with the investigated compounds at room temperature using a sitting drop vapor diffusion method. The protein (50–70 mg/mL or 0.9–1.25 mM in 100 mM potassium phosphate buffer, pH 7.4, 20% glycerol and 2 mM dithiothreitol) was incubated with a 5-fold ligand excess and centrifuged to remove the precipitate (<5% protein loss). The supernatant (0.4–0.5 μL) was mixed with 0.4–0.6 μL of the crystallization solution containing 6–10% polyethylene glycol 3350 and either 70 mM malate, 50 mM succinate, or 80 mM ammonium citrate tribasic, pH 7.0, for mibefradil-, azamulin- and DHB-bound CYP3A4, respectively. After harvesting, crystals were cryoprotected with Paratone-N and frozen in liquid nitrogen. The X-ray diffraction data were collected at the Stanford Synchrotron Radiation Lightsource beamline 7-1 and the Advanced Light Source beamline 5.0.2. The high-resolution cutoffs were chosen based on the CC1/2 value [30]. Crystal structures were solved by molecular replacement with PHASER [31], using the 5VCC structure as a search model. The ligands were built with eLBOW [32] and manually fit into the density with COOT [33]. The initial models were rebuilt and refined with COOT and PHENIX [32]. The polder and simulated annealing omit electron density maps were calculated with PHENIX. Data collection and refinement statistics are summarized in Table S1. The atomic coordinates and structure factors for mibefradil-, azamulin- and DHB-bound CYP3A4 were deposited to the Protein Data Bank with the ID codes 6OO9, 6OOA and 6OOB, respectively.

## Figures and Tables

**Table 1:** Parameters for the ligand binding to CYP3A4.

Compound	K_s_^a^ μM	High-Spin Shift	K_s_^RIT b^ μM
mibefradil	3.3 ± 0.4	65%	1.42 ± 0.03
azamulin	1.7 ± 0.3	100%	0.88 ± 0.09
bergamottin	3.0 ± 0.3 ^c^ (*n* = 1.8)	48%	0.035 ± 0.008
DHB	0.22 ± 0.03 ^d^	<3%	0.032 ± 0.002
	10.3 ± 1.4 ^d^		
bromocryptine	0.43 ± 0.08	90%	0.070 ± 0.005
midazolam	24 ± 3	85%	0.071 ± 0.006
none			0.019 ± 0.002

^a^ Spectral dissociation constant for ligand-free CYP3A4. ^b^ Binding affinity of ritonavir for ligand-bound/free CYP3A4. ^a,b^ Values represent an average of three measurements with the standard error. ^c^ S_50_; *n* is a Hill coefficient. ^d^ Dissociation constants for two binding sites.

## Supplementary Materials

Supplementary materials can be found at https://www.mdpi.com/1422-0067/20/17/4245/s1 and include five figures, showing spectral changes observed upon displacement of CYP3A4-bound bromocryptine, midazolam, bergamottin and DHB by ritonavir, simulated annealing omit electron density maps, superposition of the ligand-free and mibefradil-, DHB- and azamulin-bound structures of CYP3A4, and a table with the X-ray data collection and refinement statistics.

## Abbreviations

CYP3A4	cytochrome P450 3A4
DDI	drug-drug interaction
DHB	6′,7′-dihydroxybergamottin
MBI	mechanism-based inactivation
