# Supplementary material for: Structural Insights into the Interaction of Cytochrome P450 3A4 with Suicide Substrates: Mibefradil, Azamulin and 6′,7′-Dihydroxybergamottin
Source: Int J Mol Sci. 2019 Aug 30;20(17):4245. doi: 10.3390/ijms20174245 (PMC6747129; doi:10.3390/ijms20174245)
Supplement: Supplementary file 1 [file ijms-20-04245-s001.pdf]

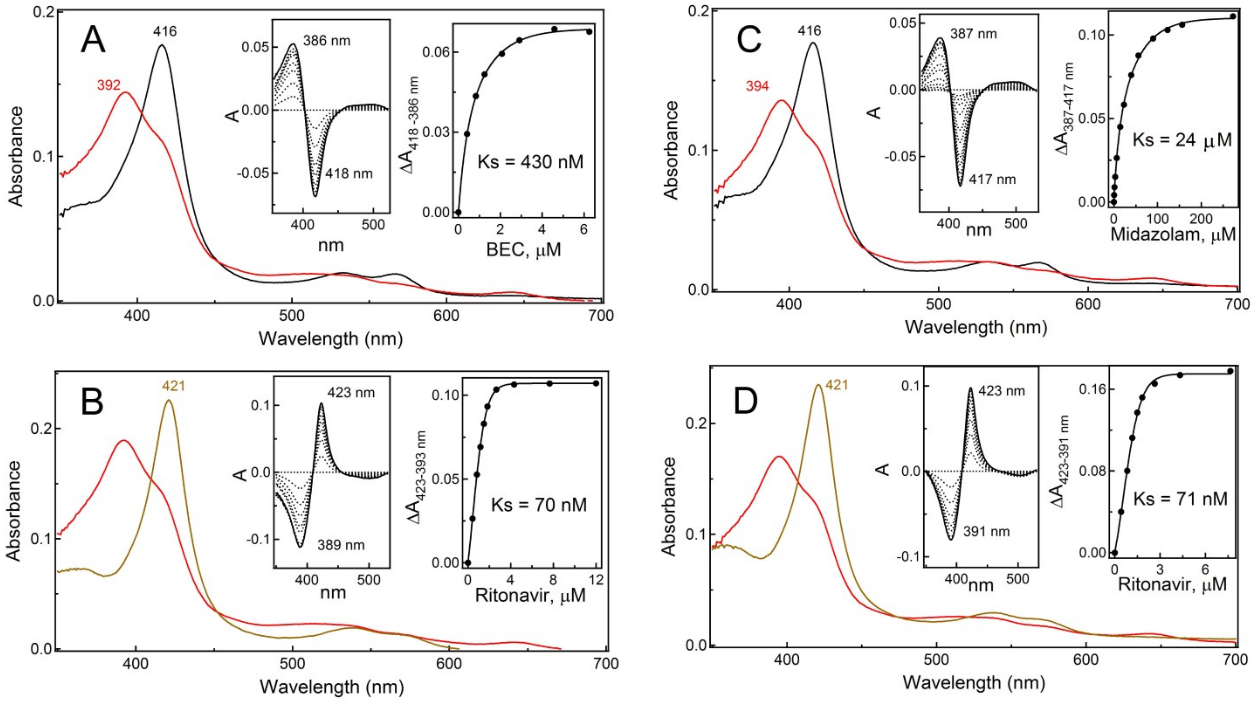

Figure S1. Spectral properties of the bromocryptine (BEC)- and midazolam-bound CYP3A4 (panels A-B and C-D, respectively). A and C – Spectral changes observed during equilibrium titrations of ligand-free CYP3A4 with bromocryptine and midazolam, respectively. B and D – Spectral changes observed during equilibrium titrations of bromocryptine- and midazolam-bound CYP3A4, respectively, with ritonavir. In panels A and C, absorbance spectra of ligand-free and substrate-bound CYP3A4 recorded at the end of titration are in black and red, respectively. In panels B and D, spectra of the CYP3A4-ritonavir complex are in light-brown. In competitive displacement experiments, the bromocryptine and midazolam concentrations were 10 μM and 280 μM, respectively. In all panels, left insets are the difference spectra recorded in a separate experiment where equal amounts of dimethyl sulfoxide (DMSO) were added to the reference cuvette to correct for the solvent-induced spectral perturbations. Right insets are titration plots derived from the difference spectra with hyperbolic or quadratic fittings. Spectral dissociation constants (K_S) are indicated.

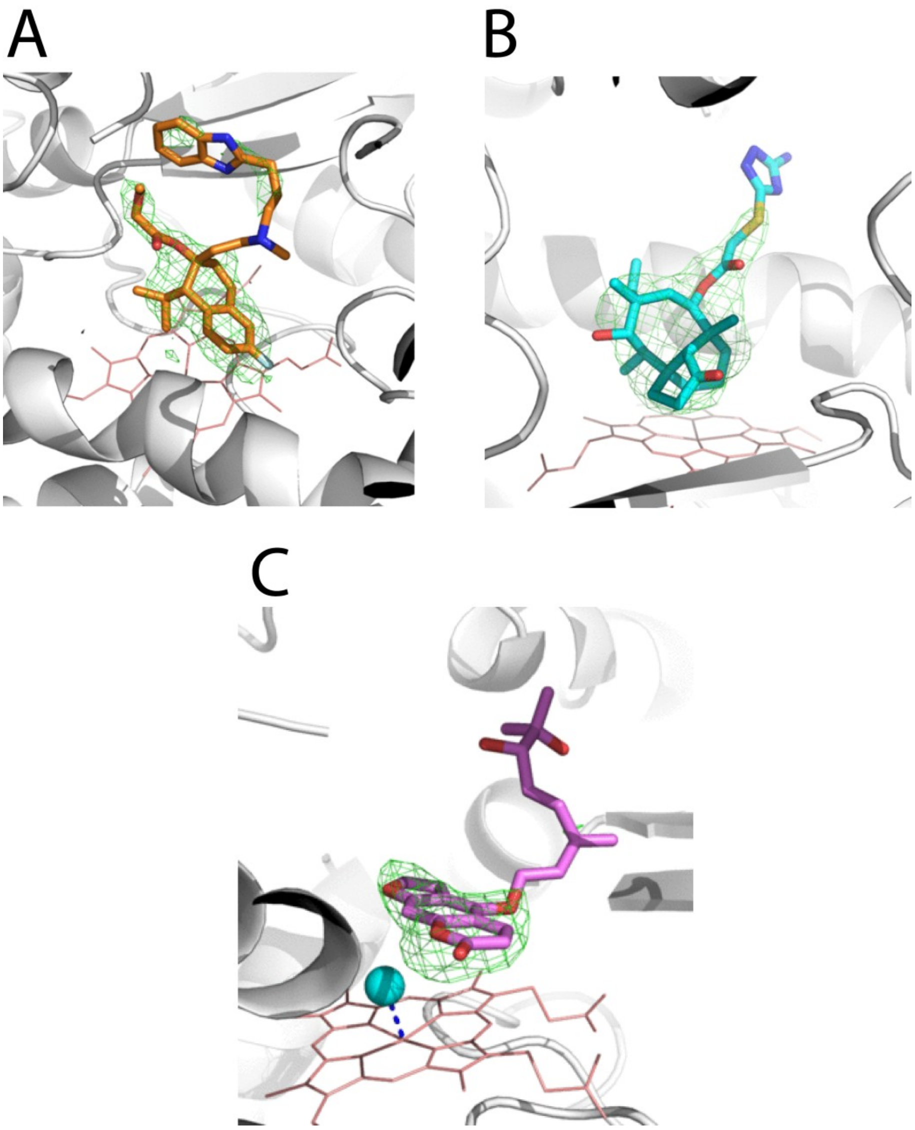

Figure S2. A-C, Simulated annealing omit electron density maps for mibefradil, azamulin and 6′,7′-dihydroxybergamottin (6OO9, 6OOA and 6OOB structures, respectively) shown as green mesh and contoured at 3σ level. In panel C, cyan sphere is a water molecule ligated to the heme iron.

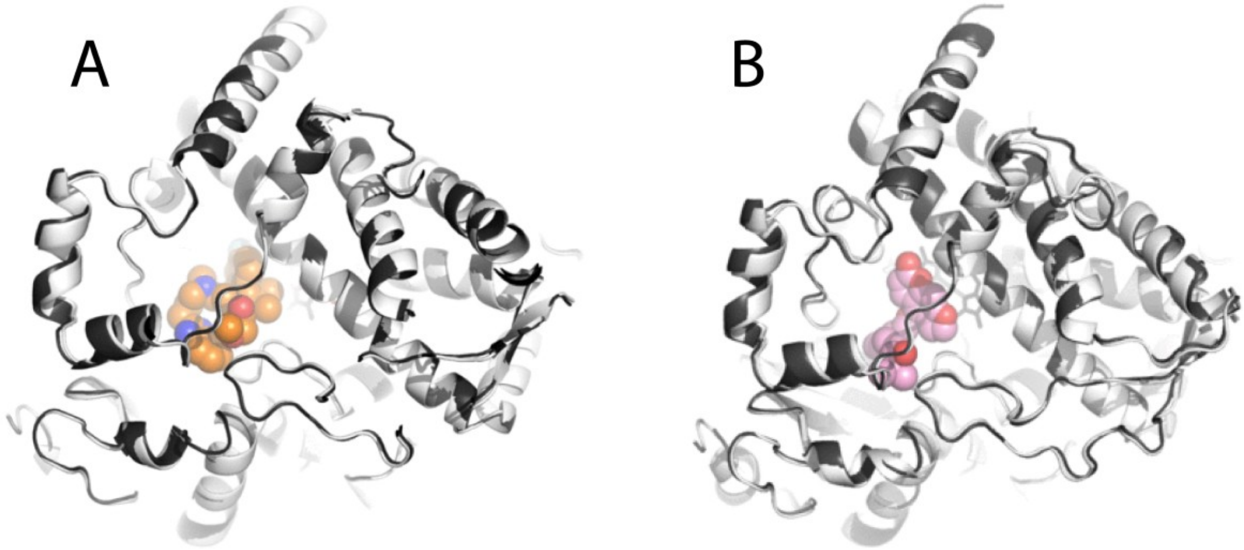

Figure S3. A and B, Structural superposition of ligand-free CYP3A4 (in black; 5VCC model) and its complexes with mibefradil and 6',7'-dihydroxybergamottin (6OO9 and 6OOB structures, respectively). Virtually no structural rearrangement was induced upon association of both substrates (shown in space-filling representation). Root-mean-square deviation between the Cα-atoms of the superimposed structures was < 0.45 Å.

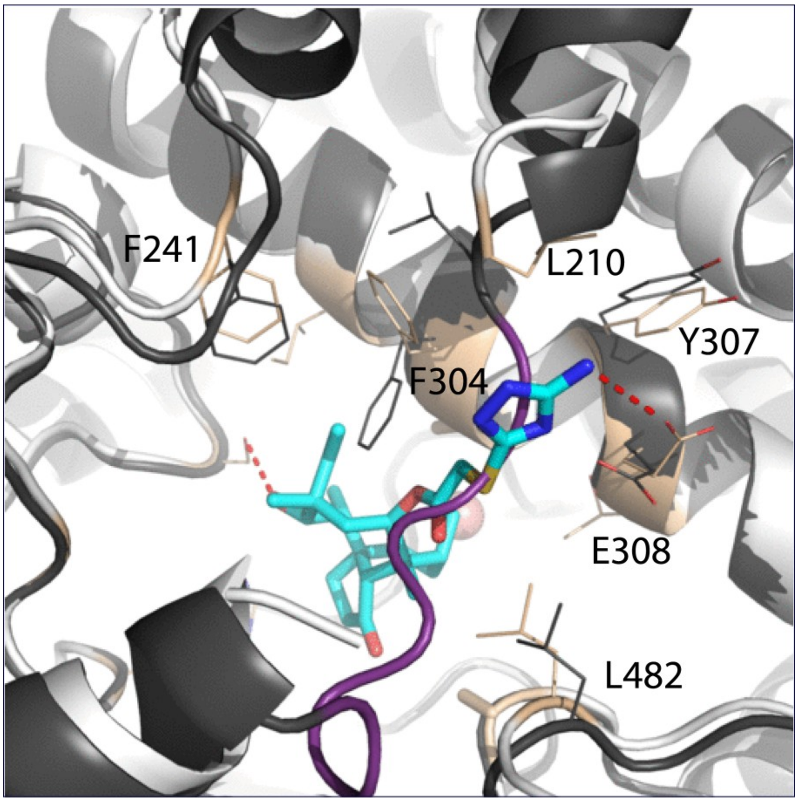

Figure S4. Superposition of the ligand-free (5VCC; in black) and azamulin-bound CYP3A4 (6OOA; in beige). Residues undergoing conformational rearrangement are displayed and labeled. The F-F' loop, shown in purple in the 5VCC structure, becomes disordered in the CYP3A4-azamulin complex due to steric clashing with the amino-triazolyl end-group. Root-mean-square deviation between the Cα-atoms of the 5VCC and 6OOA structures is 0.63 Å. .

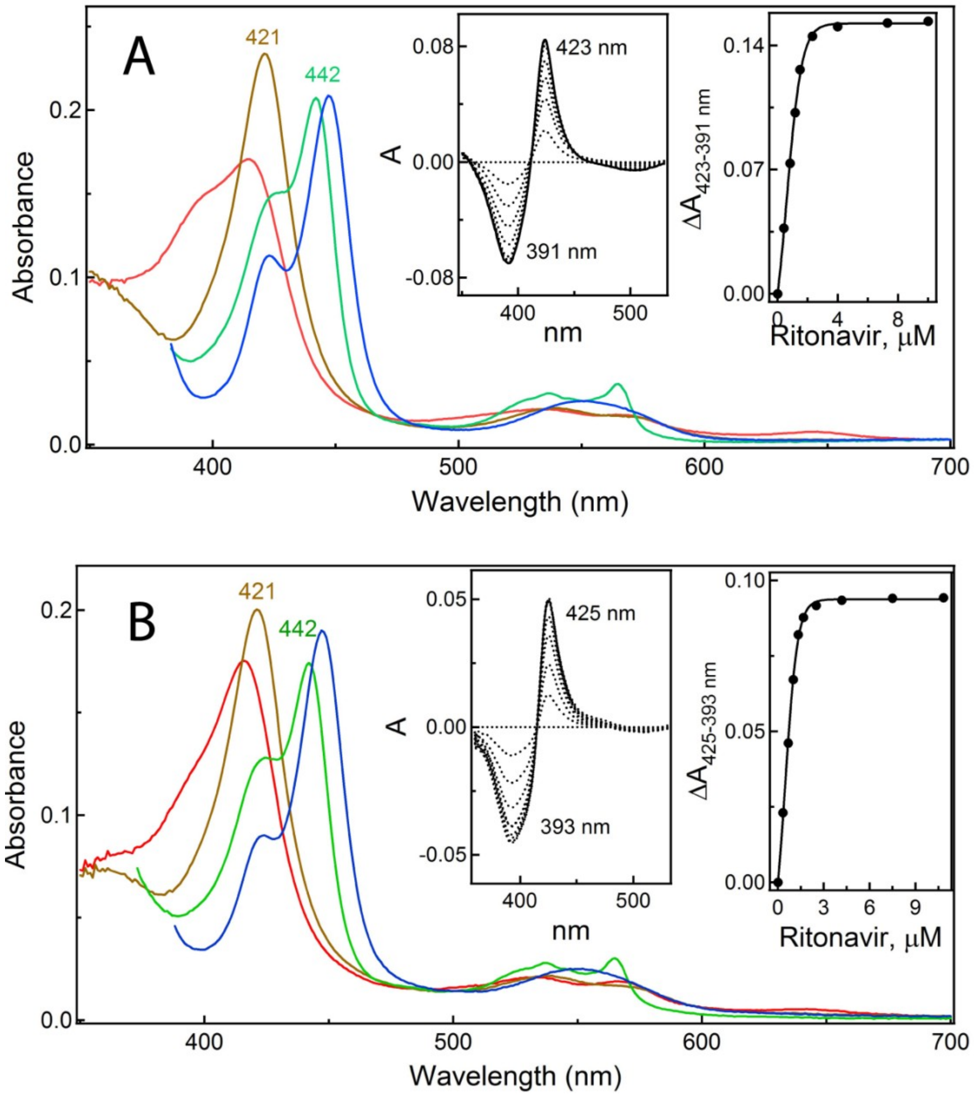

Figure S5. A and **B**, Spectral changes observed during equilibrium titrations of bergamottin- and DHB-bound CYP3A4, respectively, with ritonavir. Spectra of substrate-bound CYP3A4 are in red. Spectra of the CYP3A4-ritonavir complex and its ferrous and ferrous CO-bound forms are in brown, green and blue, respectively. Bergamottin and DHB concentrations were 20 μM and 70 μM, respectively. Left and right insets are the difference spectra and titration plots with quadratic fittings, respectively. The derived spectral dissociation constants for ritonavir (K_S^{RIT}) were similar and equal to 35 and 32 nM, respectively.

Table S1. Data collection and refinement statistics.

Ligand	mibefradil	azamulin	6',7'-dihydroxybergamottin
PDB code	6OO9	6OOA	6OOB
Data collection statistics			
Space group	I222	I222	I222
Unit cell parameters Å,	*a* = 78 Å, *b* = 103 Å, *c* = 127 Å; α, β, γ = 90°	*a* = 77 Å, *b* = 102 Å, *c* = 126 Å; α, β, γ = 90°	*a* = 78 Å, *b* = 102 Å, *c* = 127 Å; α, β, γ = 90°
Molecules per asymmetric unit	1	1	1
Resolution range (Å)	79.99-2.25 (2.37-2.25) [a]	78.97-2.52 (2.66-2.52)	79.82-2.20 (2.27-2.20)
Total reflections	120,774	94,344	196,832
Unique reflections	22,832	16,915	26,105
Redundancy	5.3 (5.1)	5.6 (5.6)	7.5 (5.2)
Completeness	93.4 (93.7)	100.0 (100.0)	99.7 (97.6)
Average $I/\sigma I$	8.7 (0.9)	11.1 (1.2)	6.8 (1.0)
R_{merge}	0.082 (1.481)	0.074 (1.502)	0.102 (0.882)
R_{pim}	0.038 (0.691)	0.034 (0.691)	0.047 (0.628)
CC ½	0.998 (0.458)	0.999 (0.366)	0.998 (0.482)
Refinement statistics			
R/R_{free} [b]	19.9/26.1	19.5/25.2	20.7/27.5
No. of protein atoms	3748	3650	3689
No. of ligand atoms	35	32	26
No. of water molecules	59	15	37
Average *B*-factor (Å^2):			
Protein	92.2	103.9	97.0
Ligand	105.4	104.4	133.9
Ligand fit:			
RSCC	0.89	0.94	0.88
RSR	0.43	0.25	0.45
r.m.s. deviations:			
Bond lengths, Å	0.009	0.009	0.009

Bond angles, °	1.129	1.159	1.123
Ramachandran plot[c] (residues; %)			
Preferred	418 (93.5%)	414 (93.9%)	418 (92%)
Allowed	29 (6.5%)	27 (6.1%)	37 (8%)
Outliers	none	none	1 (0.2%)

[a]Values in brackets are for the highest resolution shell.
[b]R_{free} was calculated from a subset of 5% of the data that were excluded during refinement.
[c]Analyzed with PROCHECK.
